# Host genetics influences HIV reservoir persistence, treatment outcomes and CCR5 expression in patients on long-term antiretroviral therapy in Ghana

**DOI:** 10.3389/fimmu.2026.1885690

**Published:** 2026-07-22

**Authors:** Peter Kwabena Appiah, Evelyn Yayra Bonney, Christian Obirikorang, James Odame Aboagye, Mildred Egyirba Hanson, Samuel Kwame Sopuruchi Agomuo, Alfred Effah, Max Efui Annani-Akollor, George Boateng Kyei

**Affiliations:** 1 Noguchi Memorial Institute of Medical Research, University of Ghana, Legon, Ghana; 2 Department of Molecular Medicine, School of Medical Sciences, Kwame Nkrumah University of Science and Technology, Kumasi, Ghana; 3 Medical and Scientific Research Centre, University of Ghana Medical Centre, Legon, Accra, Ghana; 4 Department of Medicine, Washington University School of Medicine in St Louis, St. Louis, MO, United States

**Keywords:** AIDS restriction genes, antiretroviral therapy, CCR5 polymorphisms, HIV latent reservoir, human immunodeficiency virus, SDF1-3′A

## Abstract

**Objective:**

This study investigated influence of cysteine-cysteine chemokines (CCR5, CCR5-59029A/G, CCR2), and stoma cell derived factor 1 (SDF1); as AIDS restriction genes (ARGs), on treatment outcomes among people with HIV (PWH) on long term antiretroviral therapy (ART) in Ghana.

**Methods:**

This cross-sectional study was conducted among 167 PWH and receiving ART in Accra, Ghana. Genomic DNA from PBMCs was used to genotype CCR5-Δ32, CCR2-V64I, CCR5 A59029G, and SDF1-3′A variants using polymerase chain reaction-restriction fragment length polymorphism, CCR5 expression was estimated by RT-qPCR.

**Results:**

All participants carried the wild-type CCR5 genotype. No AIDS Restriction Genes was associated with virologic or immunological outcomes (*p*>0.05). However, individuals with the CCR5 A59029G GG genotype exhibited significantly higher proviral DNA levels than those with the AA genotype (*p* < 0.05), and the GG genotype independently predicted higher proviral DNA burden in multivariable analysis (β = 0.840, *p* = 0.011).

**Conclusion:**

No ARG variant was significantly associated with virologic or immunological outcomes. However, CCR5 A59029G was associated with differences in proviral DNA burden and CCR5 mRNA expression, suggesting a potential role in HIV reservoir dynamics during suppressive ART.

## Highlights

- AIDS restriction gene variants were not associated with viral suppression or immune recovery during long-term ART in Ghana.
- CCR5 A59029G GG genotype was associated with higher proviral DNA burden and independently predicted reservoir size.
- CCR5 A59029G influenced CCR5 mRNA expression without corresponding differences in CD4 cell counts.

## Introduction

The human immunodeficiency virus (HIV) remains a significant global public health threat, with over 40.8 million people living with HIV and 1.3 million newly infected in 2024 (1). Africa has the highest prevalence, with over two thirds of people diagnosed with HIV living in Africa (1). In Ghana, the HIV prevalence in the general population is 1.6% in 2024 with regional variations (2). HIV type 1 (HIV-1) is the predominant strain responsible for the global pandemic, and it is characterized by its ability to progressively destroy the host immune system, ultimately leading to acquired immunodeficiency syndrome (AIDS) (3). The main challenge associated with HIV infection is the gradual reduction in their CD4 T-lymphocyte numbers, leading to a suppressed immune system and increased susceptibility to opportunistic infections (4, 5).

Antiretroviral therapy (ART) has transformed HIV-1 from a subacute, rapidly progressive and dangerous condition to a slowly advancing chronic disorder, leading to longer life expectancy (6, 7). However, not all individuals are able to achieve viral suppression and/or immune recovery (8). In Ghana, Opoku et al. reported a viral suppression rate of approximately 76% (9) while another study reported 68.2% (10). Both studies attributed the inability of PWH to achieve viral suppression to type of ARVs, drug resistance development, WHO clinical stage, baseline viral load, and adherence patterns, without considering the effect of host genetic variability on antiretroviral response (9, 10). For the past four decades, finding a cure has remained elusive, largely because HIV is able to develop an early reservoir of latency in infected cells, especially CD4 T-lymphocytes, which becomes transcriptionally latent and imperceptible to the host immune system and current antiretroviral regimens (11, 12).

HIV-1 gains entry into host cells using its glycoprotein 120 (gp120), which binds to CD4+ T-lymphocytes, triggering conformational changes that expose gp41 and catalyze membrane fusion (13, 14). The host cell co-receptor utilized is either CCR5 or CXCR4, making these chemokine receptors very vital in HIV infection and pathogenesis (15). HIV-1 uses CCR5 during primary infection and it is CCR5-utilising strains that are preferentially transmitted, while CXCR4-utilising strains are associated with AIDS, severe immunodeficiency, and lower CD4 counts (16, 17). The discovery of the CCR5 Δ32 allele, which is a 32 base pair deletion in the open reading frame of CCR5, demonstrated that host genetic mutations can modify HIV susceptibility and disease progression (13). In this mutant type, the truncated protein gets trapped in the endoplasmic reticulum and is unable to move to the cell membrane to be expressed, rendering the cell resistant to HIV-1 (18). Moreover, higher CCR5 density in HIV-1-infected people has been linked with higher viral loads, less responsiveness to antiretroviral therapy, and accelerated disease progression (13).

Among other well-characterized AIDS restriction genes (ARGs), CCR2-64I postpones AIDS by two to four years through an indirect mechanism involving formation of CCR2-CXCR4 heterodimers that prevent CXCR4 availability needed for the R5 to X4 tropic transition (19, 20). The SDF1-3′A mutation, caused by a G to A transition at position 801 of the SDF-1 gene, leads to increased SDF1 expression, whose binding with CXCR4 inhibits HIV gp120 interaction and thereby slows progression to AIDS (15, 21). Studies have shown that these ARGs affect the efficacy of cART in achieving viral suppression, immune response, and progression to AIDS, with variable results across settings (22, 23). Yet in Ghana, no studies have investigated the distribution of these host genetic mutations or their association with antiviral response outcomes and latent reservoir size. It is imperative to research the impact of host genetic determinants on HIV treatment outcomes and support future individualized therapeutic and cure strategies. This study therefore investigated the distribution of CCR5-Δ32, CCR2-V64I, CCR5 A59029G, and SDF1-3′A variants and their influence on virological outcomes, immunological status, and HIV latent reservoir burden among PWH on ART in Ghana.

## Methods

### Study design and sites

Participants for this cross-sectional study were randomly selected from a cohort which recruited PWH from the Korle Bu Teaching Hospital (KBTH), Ledzokuku Krowor municipality Assembly (LEKMA) hospital, the University of Ghana Hospital, Legon for the parent study. Genotyping and other experiments were carried out at Noguchi Memorial Institute for Medical Research, in the Greater Accra Region of Ghana.

### Sample size and population

This study employed a three-arm comparative design, stratifying PWH on cART into three groups based on viral load response: virally suppressed (HIV RNA <50 copies/mL), low-level viraemia (50–1,000 copies/mL), and virological failure (>1,000 copies/mL) after ≥6 months on ART. For comparing allele and genotype distributions across these three groups, the chi-square (χ²) test of independence was used. Sample size was therefore determined using *a priori* power analysis for chi-square tests, based on Cohen’s effect size w (24),

Where:

• *w* = effect size (Cohen’s w = 0.35), justified by the moderate-to-strong associations between CCR5/SDF1 polymorphisms and HIV disease outcomes reported in comparable African studies (21).

• *α* = two-sided significance level (α = 0.05).

• *df* = degrees of freedom (df = 4), derived from the 3 × 3 contingency table design.

• 1 − *β* = statistical power (0.80), indicating an 80% probability of detecting a true association if it exists.

Power analysis was conducted using G*Power software (25). The minimum sample size obtained after applying the above parameters was 115 participants. A total of 167 individuals were totally recruited to improve statistical power.

The study population was PWH who attended clinics for their routine antiretroviral medications. Written informed consent was obtained according to the declaration of Helsinki after the study processes, purpose, benefits and risks involved were explained. Individuals older than 18 years and those who were on antiretroviral treatment for more than six months were included in the study. Children living with HIV, adults with HIV who were cART naïve and eligible participants who did not provide informed consent were excluded from participation.

### Data and sample collection

After obtaining informed consent, baseline characteristics including sex, age, highest level of education, monthly income, years diagnosed with HIV, years on ART, syphilis diagnosis, regimen summary and antiretroviral adherence were obtained from participants’ medical records. Ten milliliters (10mL) of venous blood was collected from each participant into ethylenediaminetetraacetic acid (EDTA) tubes. Peripheral blood mononuclear cells (PBMCs) were isolated aliquoted and stored at -80^0^C or liquid nitrogen, whilst the plasma is stored at -40^0^Cfor further investigations.

### PBMC Isolation and Nucleic Acid Extraction

PBMCs were isolated from whole blood by Ficoll-Hypaque density gradient centrifugation. Following isolation and counting, PBMCs were aliquoted for immediate DNA and RNA extraction, with 1 × 10^6^ cells reserved for proviral DNA quantification and the remainder cryopreserved in 90% FBS/10% DMSO at −80 °C for subsequent flow cytometric analysis. DNA and RNA extractions were performed from separate PBMC aliquots washed in PBS prior to lysis, using the Qiagen DNeasy and RNeasy Plus Mini Kits respectively (QIAGEN, CA, USA) per the manufacturers’ protocols, and stored at −40 °C. Both nucleic acids were quantified on a NanoDrop One spectrophotometer (Thermo Fisher Scientific) by absorbance at 260 nm and purity was assessed by the 260/280 ratio. Samples yielding sufficient RNA for downstream analysis were taken forward for RT-qPCR.

### Genotyping of AIDS restriction gene variants

Genotyping of the four AIDS restriction gene loci was performed by polymerase chain reaction (PCR) and restriction fragment length polymorphism (PCR-RFLP). All amplifications were carried out in 25 µL reaction volumes comprising 5 µL 5× OneTaq Standard Reaction Buffer, 0.5 µL each of forward and reverse primers, 0.5 µL dNTPs, 0.125 µL Taq DNA polymerase, 5 µL template, and 13.375 µL nuclease-free water, on the AGS 4800/48 Real-Time PCR Detection System (AGTC Bioproduct Ltd., UK) without selecting the probes.

CCR5 Δ32 was detected by conventional PCR using primers CCR5-NLS-FW and CCR5-NK-RV1. Thermal cycling conditions comprised an initial denaturation at 94 °C for 30 seconds, followed by 30 cycles of 94 °C for 60 seconds, 58 °C for 60 seconds, and 68 °C for 30 seconds, with a final extension at 68 °C for 5 minutes. The wild-type and deletion alleles yielded 351 bp and 319 bp products respectively on 2% agarose gel electrophoresis.

CCR2 V64I, SDF1 3′A, and CCR5 A59029G were characterized by PCR-RFLP using previously published primers (26, 27), yielding amplicons of 380 bp, 302 bp, and 453 bp respectively. Amplicon integrity was confirmed by gel electrophoresis prior to restriction digestion. CCR2 V64I was amplified under the following cycling conditions: initial denaturation at 95 °C for 3 minutes, followed by 40 cycles of 94 °C for 30 seconds, 63 °C for 30 seconds, and 72 °C for 30 seconds, with a final extension at 72 °C for 10 minutes. SDF1 3′A and CCR5 A59029G were amplified under the same conditions as CCR5 Δ32.

Restriction digestion was performed in a 50 µL volume containing 17 µL amplicon, 27 µL nuclease-free water, 5 µL rCutSmart buffer, and 1 µL of FokI, MspI, or Bsp1286I respectively (New England BioLabs), and incubated at 37 °C for 15 minutes per the manufacturers’ instructions. The digestion patterns for CCR2-64I, SDF1 and CCR5 A59029G are summarized in **Table 1**. All digestion products were resolved on 2% agarose gels stained with GelRed nucleic acid stain and visualized under UV light.

**Table 1:** PCR and restriction digestion products for AIDS restriction gene variants.

Gene variant	PCR product size	Restriction enzyme	Wild-type	Heterozygous	Mutant
CCR5 Δ32	351 bp/319 bp	None	351 bp	351 bp + 319 bp	319 bp
CCR2 V64I	380 bp	FokI	380 bp	380 bp + 215 bp + 165 bp	215 bp + 165 bp
SDF1 3′A	302 bp	MspI	202 bp + 100 bp	302 bp + 202 bp + 100 bp	302 bp
CCR5 A59029G	453 bp	Bsp1286I	453	453 bp + 408 bp + 45 bp	408 + 45 bp

### Measurement of CCR5 mRNA expression, viral load and latent reservoir

Expression of CCR5 mRNA was quantified by RT-qPCR. Extracted RNA was reverse transcribed to cDNA using the Quantabio qScript cDNA Synthesis Kit in a 20 µL reaction per the manufacturer’s protocol. The thermal profile comprised 22 °C for 5 minutes, 42 °C for 30 minutes, and 85 °C for 5 minutes, followed by a 4 °C hold. Real-time PCR was carried out in a 20 µL reaction containing 10 µL 2× qPCR Bio Probe Blue Mix with separate ROX (PCR Biosystems Ltd., London), CCR5-NLS-FW and CCR5-NK-RV1 primers at 400 nM, and a FAM/ZEN/IBFQ-labelled probe at 200 nM (Integrated DNA Technology, Iowa, USA). GAPDH was co-amplified as a housekeeping reference gene to be used in calculating the relative gene expression of CCR5. Thermal cycling comprised an initial denaturation at 95 °C for 2 minutes, followed by 40 cycles of 95 °C for 5 seconds and 60 °C for 30 seconds. All reactions were run in duplicate on the QuantStudio™ 7 Flex Real-Time PCR system.

HIV viral load was measured on the fully automated Cobas 6800 system (Roche Molecular Diagnostics) following the WHO-prequalified standard operating procedure (PQDx 0365-046-00). The HIV latent reservoir was estimated by total proviral DNA quantification. Cryopreserved PBMCs were thawed, counted, and 1 × 10^6^ cells used for total nucleic acid extraction with the RADI Prep DNA/RNA Kit per the manufacturer’s protocol. Eluates were stored at −80 °C until use. TaqMan real-time PCR targeting the HIV-gag gene was performed using Malnati primers (Forward: TCTCGACGCAGGACTCG; Reverse: TACTGACGCTCTCGCACC) and a FAM-labelled probe on the QuantStudio 7 Pro system (Applied Biosystems). Thermal cycling conditions were 95 °C for 10 minutes, followed by 40 cycles of 95 °C for 15 seconds and 60 °C for 1 minute. Quantification was against a six-point standard curve generated from 1:5 serial dilutions of human genomic DNA starting at 10 ng/µL.

### Statistical analysis

Collected data were entered, cleaned and coded using Microsoft Excel 2019. Statistical analyses were performed using Statistical Package for Social Sciences (SPSS) Version 26.0 and R programming language version 4.4.4. Categorical variables were presented as frequencies and percentages. The chi-square test was used to compare the allele frequencies for the different ARGs. Association between ARGs and virologic outcomes, immunological outcomes, and latent reservoir status were performed using the Chi-square test. To account for multiple comparisons across categorical outcome analyses, Bonferroni-adjusted *p*-values were calculated and reported alongside raw *p*-values. Comparison of CD4 count, proviral DNA and CCR5 mRNA expression among ARGs gene variants was done by the Mann–Whitney U test or Kruskal–Wallis test. Where significant differences were identified, pairwise *post hoc* comparisons were performed to determine the specific genotype groups contributing to the observed differences. Multivariable linear regression analysis was conducted to identify independent predictors of proviral DNA levels while adjusting for ART duration, ART adherence, and viral load. *p* values less than 0.05 were considered statistically significant.

## Results

### Socio-demographic characteristics of study participants

Of the 167 participants included in the study, most were female (64.7%). The largest age group was 40–49 years (37.1%), followed by those aged 50–59 years (32.3%). Most participants had completed JHS/SHS education (61.2%), with 17% reporting no formal education. Majority of the participants reported a monthly income below 1000 Ghana cedis (70.9%), while 17.6% reported having no income (**Table 2**).

**Table 2:** Socio-demographic characteristics of study participants.

Variable	Frequency (n=167)	Percentage (%)
Sex		
Male	59	35.3
Female	108	64.7
Age group		
<30	9	5.4
30 to 39	31	18.6
40 to 49	62	37.1
50 to 59	54	32.3
60 and above	11	6.6
Highest level of education		
No formal education	28	17
Primary	16	9.7
JHS/SHS	101	61.2
Tertiary	20	12.1
Monthly income		
<1000	117	70.9
>1000	19	11.5
None	29	17.6

Data presented as frequency and percentage; participants with incomplete responses were excluded from the analysis.

### Clinical and viral characteristics

**Table 3** summarizes the clinical and viral characteristics of study participants. Most of participants of this study had been diagnosed with HIV for 6–10 years (60%) and had been on ART for more than 6 years (52.1%). Nearly all participants were TPAb negative (89.7%). DTG-based regimens were the most common (73.5%), with antiretroviral adherence reported by 32.9% of participants. Viral suppression was observed in 73.9% of the study participants, with 18.8% showing low-level viremia and 7.2% meeting criteria for virologic failure. Regarding the latent reservoir, 49.1% had medium reservoir levels, while 20.7% had high levels. Most participants were classified as optimal for immune recovery (71.6%), with smaller proportions showing moderate (26.1%) or severe (2.2%) immunosuppression.

**Table 3:** Clinical and viral characteristics.

Variable	Frequency (n=167)	Percentage (%)
Years diagnosed with HIV		
<2	17	10.3
6 to 10	99	60
11 to 19	45	27.3
20 to 30	4	2.4
Years on ART		
<2 years	31	18.8
2–6 years	46	27.9
>6 years	86	52.1
No treatment	2	1.2
TPAb		
Negative	122	89.7
Positive	14	10.3
Regimen summary		
DTG based	100	73.5
EFV based	28	20.6
NVP based	8	5.9
Antiretroviral Adherence		
No	59	35.3
Yes	55	32.9
Viral suppression		
Suppressed	102	73.9
Low level viremia	26	18.8
Virologic failure	10	7.2
Latent Reservoir		
Low	35	30.2
Medium	57	49.1
High	24	20.7
Immunosuppression		
Optimal	96	71.6
Moderate	35	26.1
Severe	3	2.2

Data presented as frequency and percentage; participants with incomplete responses were excluded from the analysis. Viral suppression was categorized as suppressed, low level viremia, and virologic failure based on Viral Load (copies/Ml) (Virally Suppressed: <50, Low-Level Viremia: 50–1000, Virologic Failure: ≥ 1000). Immunosuppression was categorized as optimal, moderate or severe based on CD4 count (cells/µL) (Optimal: ≥500, Moderate 200-499, High <200). Latent reservoir levels were categorized as low, medium, or high based on Log proviral DNA copies per 10⁶ PBMCs (Low: <2.0, Moderate 2.0-3.0, High >3.0).

HIV, Human Immunodeficiency Virus; ART, Antiretroviral Therapy; TPAb, Treponema pallidum Antibody; DTG, Dolutegravir; EFV, Efavirenz; NVP, Nevirapine.

### Association between ARGs and virologic outcomes

In this study, the CCR5 Δ32 heterozygous (WT/Δ32) and homozygous (Δ32/Δ32) genotypes were not detected in the study population, while the SDF1 3′A, AA homozygous genotype was also absent. No significant associations were observed between ARG genotypes and virologic outcomes. CCR5 Δ32 (rs333), SDF1 3′A (rs1801157), CCR5 A59029G (rs1799987), and CCR2 V64I (rs1799864) was not significantly associated with the distribution of viral suppression, low-level viremia, or virologic failure (*p* > 0.05) (**Table 4**).

**Table 4:** Association between ARGs and virologic outcomes.

		Viral suppression				
Variant	Total (n=137)	Suppressed (n=101)	Low level viremia (n=26)	Virologic failure (n=10)	*p-*value (raw)	*p*-value (Bonferroni)
CCR5 Δ32 (rs333)						
WT/WT	137 (100.0)	101 (100.0)	26 (100.0)	10 (100.0)		
WT/Δ32	–	–	–	–		
Δ32/Δ32	–	–	–	–		
**SDF1 3′A (rs1801157)**					0.822	1.000
GG	122 (93.1)	91 (92.9)	22 (95.7)	9 (90.0)		
AG	9 (6.9)	7 (7.1)	1 (4.3)	1 (10.0)		
AA	–	–	–	–		
**CCR5 A59029G (rs1799987)**					0.151	1.000
AA	58 (42.3)	48 (47.1)	6 (24.0)	4 (40.0)		
AG	47 (34.3)	35 (34.3)	9 (36.0)	3 (30.0)		
GG	32 (23.4)	19 (18.6)	10 (40.0)	3 (30.0)		
**CCR2 V64I (rs1799864)**					0.174	1.000
VV	94 (68.1)	73 (71.6)	14 (53.8)	7 (70.0)		
VI	22 (15.9)	15 (14.7)	7 (26.9)	0 (0.0)		
II	22 (15.9)	14 (13.7)	5 (19.2)	3 (30.0)		

Data are presented as frequency and percentage unless otherwise stated; participants with incomplete responses were excluded from the analysis. Viral suppression was categorized as suppressed, low level viremia, and virologic failure based on Viral Load (copies/mL) (Virally Suppressed: <50, Low-Level Viremia: 50–1000, Virologic Failure: ≥ 1000). *P*-values were calculated using the Chi-square test. *P* < 0.05 was bolded and considered statistically significant. ARG, AIDS Restriction Gene.

### Association between ARGs and immunological outcomes

After a chi-square test analysis, ARG genotypes were not significantly associated with immunosuppression status. Optimal immune recovery, moderate, and severe immunosuppression were distributed similarly across CCR5 Δ32, SDF1 3′A, CCR5 A59029G, and CCR2 V64I genotypes (*p* > 0.05) (**Table 5**).

**Table 5:** Association between ARGs and immunological outcomes.

		Immunosuppression				
Variant	Total (n=133)	Optimal (n=96)	Moderate (n=34)	Severe (n=3)	*p-*value (raw)	*p-*value (Bonferroni)
**CCR5 Δ32 (rs333)**					–	–
WT/WT	133 (100)	96 (100.0)	34 (100.0)	3 (100.0)		
WT/Δ32	–	–	–	–		
Δ32/Δ32	–	–	–	–		
**SDF1 3′A (rs1801157)**					0.781	1.000
GG	118 (92.2)	84 (91.3)	31 (93.9)	3 (100.0)		
AG	10 (7.8)	8 (8.7)	2 (6.1)	0 (0.0)		
AA	–	–	–	–		
**CCR5 A59029G (rs1799987)**					0.358	1.000
AA	55 (41.4)	41 (43.2)	14 (40.0)	0 (0.0)		
AG	46 (34.6)	29 (30.5)	15 (42.9)	2 (66.7)		
GG	32 (24.1)	25 (26.3)	6 (17.1)	1 (33.3)		
**CCR2 V64I (rs1799864)**					0.815	1.000
VV	89 (66.9)	63 (66.3)	24 (68.6)	2 (66.7)		
VI	21 (15.8)	14 (14.7)	6 (17.1)	1 (33.3)		
II	23 (17.3)	18 (18.9)	5 (14.3)	0 (0.0)		

Data are presented as frequency and percentage unless otherwise stated; participants with incomplete responses were excluded from the analysis. Immunosuppression was categorized as optimal, moderate or severe based on CD4 count (cells/µL) (Optimal: ≥500, Moderate 200-499, High <200). *p*-values were calculated using the Chi-square test. *P* < 0.05 was bolded and considered statistically significant. ARG, AIDS Restriction Gene.

### Association between ARGs and latent reservoir status

For association between ARGs and Latent Reservoir Status, SDF1 3′A (rs1801157) demonstrated a statistically significant association with latent reservoir levels (*p* = 0.046). However, after Bonferroni correction, this association lost its significance (*p* > 0.05). Similarly, CCR5 Δ32, CCR5 A59029G, and CCR2 V64I showed no significant associations with latent reservoir status (*p* > 0.05) (**Table 6**).

**Table 6:** Association between ARGs and latent reservoir status.

		Latent reservoir				
Variant	Total (n=115)	Low (n=35)	Medium (n=56)	High (n=24)	*p-*value (raw)	*p-*value (Bonferroni)
**CCR5 Δ32 (rs333)**					–	–
WT/WT	115 (100.0)	35 (100.0)	56 (100.0)	24 (100.0)		
WT/Δ32	–	–	–	–		
Δ32/Δ32	–	–	–	–		
**SDF1 3′A (rs1801157)**					**0.046**	0.410
GG	104 (93.7)	31 (93.9)	53 (98.1)	20 (83.3)		
AG	7 (6.3)	2 (6.1)	1 (1.9)	4 (16.7)		
AA	–	–	–	–		
**CCR5 A59029G (rs1799987)**					0.517	1.000
AA	49 (42.6)	17 (48.6)	24 (42.9)	8 (33.3)		
AG	39 (33.9)	13 (37.1)	18 (32.1)	8 (33.3)		
GG	27 (23.5)	5 (14.3)	14 (25.0)	8 (33.3)		
**CCR2 V64I (rs1799864)**					0.502	1.000
VV	79 (68.7)	25 (71.4)	37 (66.1)	17 (70.8)		
VI	18 (15.7)	6 (17.1)	7 (12.5)	5 (20.8)		
II	18 (15.7)	4 (11.4)	12 (21.4)	2 (8.3)		

Data are presented as frequency and percentage unless otherwise stated; participants with incomplete responses were excluded from the analysis. Latent reservoir levels were categorized as low, medium, or high based on Log proviral DNA copies per 10⁶ PBMCs (Low: <2.0, Moderate 2.0-3.0, High >3.0). *p*-values were calculated using the Chi-square test. *P* < 0.05 was bolded and considered statistically significant. ARG, AIDS Restriction Gene; PBMC, Peripheral Blood Mononuclear Cell.

### Independent predictors of HIV latent reservoir

Multivariable linear regression models were used to identify the predictors of HIV latent reservoir, adjusting for ART duration, self-reported ART adherence, and viral load. In the SDF1 3′A model, the AG genotype was not independently associated with proviral DNA levels compared to the GG reference group (β = −0.407, *p* = 0.459). Similarly, in the CCR2 V64I model, neither the II nor VI genotype was independently associated with proviral DNA levels (*p* = 0.241 and *p* = 0.664 respectively).

In the CCR5 A59029G model, the GG genotype was independently associated with significantly higher proviral DNA levels compared to the AA reference group (β = 0.840, *p* = 0.011), while the AG genotype was not (β = 0.245, *p* = 0.428). ART non-adherence emerged a significant predictor (β = 0.619, *p* = 0.028).) (**Table 7**).

**Table 7:** Independent predictors of HIV latent reservoir.

Genotype	Predictor	β	SE	*p* value
**SDF1 3′A**	SDF1 AG vs GG	-0.407	0.546	0.459
	ART duration	0.001	0.062	0.993
	ART adherence	0.609	0.294	**0.043**
	Viral load	0.122	0.116	0.294
Model: R² = 0.105, *p* = 0.194				
**CCR5 A59029G**	CCR5 GG vs AA	0.840	0.319	**0.011**
	CCR5 AG vs AA	0.245	0.306	0.428
	ART duration	-0.019	0.057	0.736
	ART adherence	0.619	0.273	**0.028**
	Viral load	0.041	0.111	0.716
Model: R² = 0.202, *p* = 0.026				
**CCR2 V64I**	CCR2 II vs VV	-0.442	0.373	0.241
	CCR2 VI vs VV	-0.157	0.360	0.664
	ART duration	-0.014	0.059	0.815
	ART adherence	0.553	0.281	0.053
	Viral load	0.134	0.113	0.240
Model: R² = 0.123, *p* = 0.183				

Bolded p-values indicate statistical significance.

### Comparison of CD4 Count, Proviral DNA and CCR5 mRNA expression among ARGs gene variants

**Figure 1:**
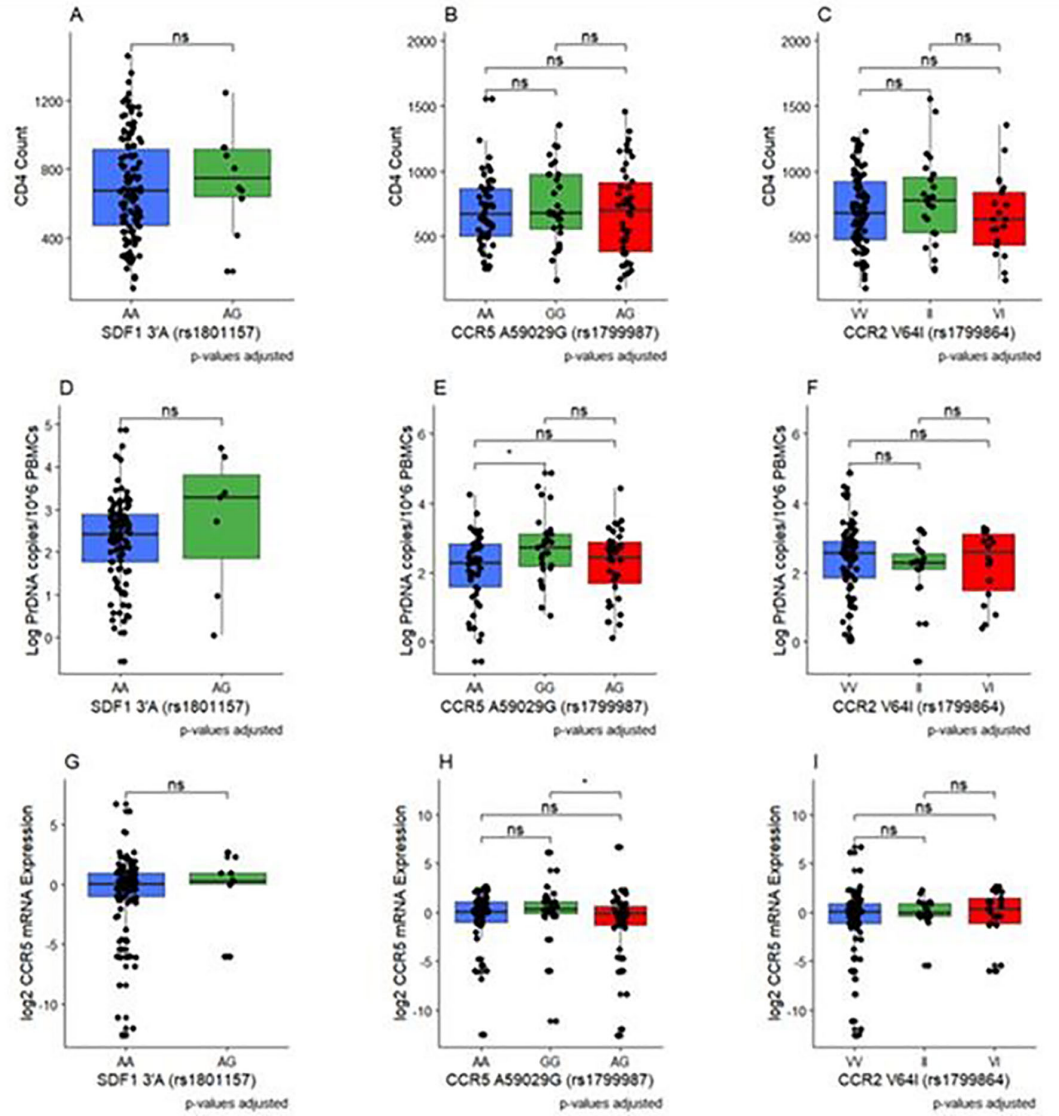
Comparison of CD4 Count, Proviral DNA and CCR5 mRNA Expression among ARGs Gene Variant. Box and whiskers plot showing **(A–C)**; the relationship between CD4 count and the various genotypes. **(D–F)**; The relationship between proviral DNA and genotypes and **(G–I)** the relationship between CCR5 mRNA expression and the various genotypes. Kruskal Wallis test p-values were presented, p < 0.05 was considered statistically significant. ns: non-significant.

The comparative analysis of CD4 count, proviral DNA levels, and CCR5 mRNA expression across allele variants is presented in **Figure 1**. A significant difference in proviral DNA levels was observed between the AA and GG genotypes of CCR5 A59029G (rs1799987) (*p* < 0.05). Similarly, CCR5 mRNA expression differed significantly between the GG and AG genotypes of the same variant (*p* < 0.05).

However, CD4 count showed no significant differences across genotypes of SDF1 3′A (rs1801157), CCR5 A59029G (rs1799987), or CCR2 V64I (rs1799864). Proviral DNA levels did not differ significantly across any other genotypes, and CCR5 mRNA expression likewise showed no additional significant variation across genotype groups.

## Discussion

This study investigated the association of cysteine-cysteine chemokine receptor gene variants and SDF1/SDF1-3A polymorphisms with treatment outcomes and HIV latent reservoir size among PWH on long term ART in Ghana. The genotypic distributions of ARGs in this study among PWH on cART shows the complete absence of the CCR5-Δ32 mutation. This deletion is in line with a body of literature indicating that the deletion is virtually non-existent in sub-Saharan Africa. Investigations and studies on Nigerian and Zimbabwean populations found 0% prevalence (28), which is similar to Angolan, Kenyan populations, and other African populations (29, 30), a recent study in Cameroon however found the Δ32 in the heterozygous state in some individuals in Northen Cameroon (31), which happens to be the only finding of Δ32 in Africa so far. The wild-type dominance acts as an additional factor in increased HIV vulnerability without the partial protection heterozygosity provides to Europeans (29, 30). The Δ32 deletion that confers resistance to HIV-1 by inhibiting the expression of functional CCR5 is present at sufficiently high frequencies only in the populations of Europe, with allele frequencies reaching 10-15% in Northern Europe (29). Its absence in this study indicates the minimal role of the protective variant in the epidemiology of HIV on the African continent where HIV burden is the greatest. It also probably contributes to the difficulties in developing viral suppression and control latent reservoirs because CCR5-Δ32 heterozygotes in other populations have slower progression and reduced reservoir. Furthermore, the lack of such mutation highlights the significance of other ARGs in regulating disease outcome among African populations.

The lack of marked correlations between these AIDS restriction gene (ARG) polymorphisms and virologic or immunological phenotypes in this study highlights the complexity of host-virus interactions during ART. Although genetic variants like CCR5-D32, CCR5 A59029G and CCR2-V64I have been linked to HIV infection and progression in other populations, their role in long-term suppressive therapy seems insignificant. As for example CCR5 A59029G variant G/G genotype was more frequently found among PWH in Cameroon (34.3%) compared to those without HIV (15.6%), indicating an increment in infection susceptibility through amplified promoter activity (21). Similarly, SDF1-3’A and CCR2-V64I did not express any significant associations with virologic and immunological outcomes. Such polymorphisms displayed incongruent and mostly non-significant relationships with HIV-1 disease progression in various populations. Although previous researchers proposed small protective effects, these arguments have been refuted by the later studies. Wit and colleagues did not observe significant variations in viral clearance or response to treatment in patients with and without such polymorphisms (32). In another study, it was reported that effects of treatment probably overcome genetic contributions (33), while Farissi et al. did not detect any significant association with immunovirological outcomes (34). This heterogeneity is probably caused by individual population genetics and the over-imposing effects of antiretroviral therapy in the control of HIV disease.

Although the unadjusted analysis suggested an association between SDF1-3’A and latent reservoir size, this relationship did not remain significant after Bonferroni correction for multiple comparisons. The high prevalence of the AG genotype in participants with high reservoirs (16.7%), compared with the predominance of the GG genotype in low (93.9%) and medium (98.1%) reservoir groups, may suggest a potential relationship between SDF1 variation and reservoir dynamics, which is also demonstrated by the visibly wider spread and higher median in the AG compare to the GG in **Figure 1D**. However, the very small number of AG carriers included in the latent reservoir analysis limits the robustness of this observation. SDF1 gene codes stromal cell-derived factor 1 (CXCL12), the natural ligand of CXCR4, which is a co-receptor of HIV entry (35). The 3′A variant has been linked to slower HIV progression and CD4 count decline in various populations and across study designs (23, 36). The trend seen might be due to modified signaling through CXCR4, modulating the ability of long-lived CD4+ T cells to become or remain latently infected (37). Nevertheless, the loss of statistical significance following correction for multiple testing, together with the small size of the subgroup, does not support an independent association between SDF1-3’A and HIV reservoir size. The finding remains preliminary and warrants assessment in larger, adequately powered cohorts that include more carriers of the variant genotype.

The comparative analysis of the level of CD4, proviral DNA and CCR5 mRNA expression among ARG variants indicated significant variation in CCR5 A59029G (rs1799987). In particular, there were significantly more elevated levels of proviral DNA in the GG than the AA genotypes. Also, significantly reduced CCR5 mRNA was found in the AG genotype in comparison with the GG. This observation supports earlier findings that promoter SNPs may have either functional or non-functional effects on reservoir modulation and receptor regulation (38). CCR5 A59029G variant may act as a reservoir modulator, which is most probably due to differences in expression, affecting proviral integration (38). The observation that the proviral DNA concentration of GG carriers was much greater than that of AA carriers is consistent with earlier findings that CCR5 promoter polymorphisms were shown to modulate receptor density and viral entry capacity (39). The G allele has been linked to changes in transcriptional activity and receptor expression (40).

The increased proviral DNA burden seen in GG carriers was not accompanied by differences in CD4 cell counts. That pattern suggests that the effect of this polymorphism may be more specific to reservoir dynamics than to overall immune recovery. Variation at the CCR5 locus has implications that extend beyond CCR5 expression and viral entry. Effects on the establishment and persistence of infected cellular clones are also conceivable. HIV persistence during suppressive ART is understood to result largely from the clonal expansion of infected CD4^+^ T cells rather than from ongoing viral replication (41, 42). Against this background, the CCR5 A59029G GG genotype may favor the survival or expansion of infected cells and thereby contribute to higher proviral DNA levels, despite comparable CD4 recovery. Although our study did not directly assess integration sites or clonal expansion, these mechanisms warrant investigation in future studies.

While studies have described that the G allele increases the transcriptional activity of CCR5 (40), another mentioned that the CCR5 promoter 59029G allele is associated with low transcriptional activity and, as a result, low CCR5 expression in comparison with the CCR5 promoter 59029A allele (43). These contrasting results reveal the intricacy of CCR5 regulation, which can differ among populations, made possible by other post translational modifications that affect CCR5 expression and availability, such as sulfation palmitoylation and lipid raft availability on the host cell membrane which all influence CCR5 expression (44) The high proviral DNA concentrations in GG carriers reported in this present study may thus represent population-specific regulatory processes, in which decreased CCR5 transcription does not always result in decreased reservoir seeding, potentially because of compensatory processes in immune activation or viral tropism and post translational modification (44, 45). The functional impact of this polymorphism is also supported by the observed differences in the CCR5 mRNA expression of the GG and AG genotypes. The increase in CCR5 in GG carriers is in line with findings that the G allele can amplify the promoter activity, and the smaller expression of CCR5 in AG heterozygotes than GG carriers indicate allele-specific regulatory interactions (40). Notably, it is reported that CCR5 expression is directly related to the susceptibility of target cells to HIV-1 infection, and the density of CCR5 on CD4+ T cells predisposes individuals to infection (46). CCR5 promoter 59029 A/G polymorphism is thus an essential factor of host predisposition, where the promoter affects the expression of the receptor and the efficiency of viral infiltration (40).

There was no significant difference in the number of CD4 cells, proviral DNA, or CCR5 expression in SDF1-3’A and CCR2 V64I genotypes. This supports the inconsistent findings in previous studies carried out on these variants. While a study in Brazil found no significant differences in the biological markers among these genotypes (23), another study in Iran demonstrated that CCR2 V64I was linked to reduced risk of AIDS progression (45), and yet another study revealed that the genetic variations have little role to play in natural history of HIV-1 infection (47). CCR2-64I mutation is capable of affecting the level of CD4 and HIV-1 RNA but its cumulative effect on disease progression is relatively small (48). These findings highlight that these genetic variants have subtle, population specific effects rather than severe clinical implications.

Likewise, SDF1-3’A has been inconsistently associated with disease progression. The SDF1-3’A allele carriers showed higher rates of reduction in the count of CD4+ T cells, with regional differences demonstrated with positive associations in Caucasian and Asian long-term non-progressors (49). A previous global meta-study concluded that SDF1-3’A homozygotes did not have any significant protective effect (36), underscoring the complexity of the influence of this genetic variant on HIV progression.

Although this study fills an important gap by investigating ARGs in relation to reservoir persistence in Ghana, few limitations are worth acknowledging. The cross-sectional design limits inference regarding causality between host genetic variants and reservoir dynamics. The low prevalence, and in some cases absence, of particular genotypes, especially CCR5 Δ32 and SDF1 3′A homozygotes, may have constrained the detection of genotype-specific effects. Reservoir estimates were derived from total HIV proviral DNA, which encompasses defective and intact proviruses and may therefore overestimate the size of the replication-competent reservoir. Although total HIV DNA is widely accepted as a surrogate measure of reservoir size, intact proviral DNA assays and viral outgrowth assays would provide a more precise evaluation of the rebound-capable reservoir.

## Conclusion

Our study found that SDF1 3′A showed a statistically significant association with high latent reservoir levels, and CCR5 A59029G, specifically the GG genotype influenced proviral DNA load and CCR5 mRNA expression.

However, none of the AIDS restriction gene variants was associated with virologic or immunological treatment outcomes, though small or population-specific effects cannot be ruled out due to the limited sample size studied. No association observed with virologic suppression suggests that once on effective ART, host genetic variation in entry co-receptors does not appear to play a major role in viral control and thus, host genetic characteristics may not substantially influence whether ART reduces the virus in circulation but rather the size and durability of the latent HIV reservoir, this will require further studies with a larger sample size as it will be crucial for HIV cure strategies.

## Data Availability

The raw data supporting the conclusions of this article will be made available by the authors, without undue reservation.
